# A computational method for detecting copy number variations using scale-space filtering

**DOI:** 10.1186/1471-2105-14-57

**Published:** 2013-02-18

**Authors:** Jongkeun Lee, Unjoo Lee, Baeksop Kim, Jeehee Yoon

**Affiliations:** 1 Cancer Genomics Branch and Research Institute and Hospital, National Cancer Center, Goyang, Korea; 2 Department of Computer Engineering, Hallym University, Chuncheon, Korea; 3 Department of Electronic Engineering, Hallym University, Chuncheon, Korea

## Abstract

**Background:**

As next-generation sequencing technology made rapid and cost-effective sequencing available, the importance of computational approaches in finding and analyzing copy number variations (CNVs) has been amplified. Furthermore, most genome projects need to accurately analyze sequences with fairly low-coverage read data. It is urgently needed to develop a method to detect the exact types and locations of CNVs from low coverage read data.

**Results:**

Here, we propose a new CNV detection method, CNV_SS, which uses scale-space filtering. The scale-space filtering is evaluated by applying to the read coverage data the Gaussian convolution for various scales according to a given scaling parameter. Next, by differentiating twice and finding zero-crossing points, inflection points of scale-space filtered read coverage data are calculated per scale. Then, the types and the exact locations of CNVs are obtained by analyzing the finger print map, the contours of zero-crossing points for various scales.

**Conclusions:**

The performance of CNV_SS showed that FNR and FPR stay in the range of 1.27% to 2.43% and 1.14% to 2.44%, respectively, even at a relatively low coverage (0.5x ≤C ≤2x). CNV_SS gave also much more effective results than the conventional methods in the evaluation of FNR, at 3.82% at least and 76.97% at most even when the coverage level of read data is low. CNV_SS source code is freely available from http://dblab.hallym.ac.kr/CNV SS/.

## Background

After the findings of Down syndrome in the 1950s, the relevance between copy number variation (CNV) and genetic disorders has been extensively researched, and many studies have found Crohn’s disease, type 1 diabetes, rheumatoid arthritis, and mental and development disorders to be connected with CNV [1]. Furthermore, as next-generation sequencing (NGS) technology became more widely used, the genome-wide association study accelerated, hence amplifying the importance of finding CNVs related to diseases and phenotypes. The connection of CNVs found in the Long QT syndrome genes KCNQ1 and KCNQ2 has recently been stated [2], and primary open-angle glaucome has been found to be connected with 11 rare noble CNVs [3]. Additionally, CNVs related to attention deficit hyperactivity disorder and schizophrenia have been concluded to show disease-specific patterns [4,5]. These studies indicate the importance of CNV detection and analysis, especially in the personalized medicine that is based on race-specific or disease-specific genetic expressions.

Since 2008, researchers in 75 colleges and companies worldwide, through a consortium called the 1000 Genome Project, have found 95% of the diversity in the human genome, as well as 15 million genetic diversities that had not been studied before [6]. Many genome projects are actively being carried out around the world, individually or by forming consortiums, to analyze genomic sequences not only of humans, but also of other living organisms (http://ldl.genomics.cn; http://genome10k.soe.ucsc.edu/). Therefore, it is expected that a massive amount of data regarding various genetic sequences is being produced, resulting in the urgent need to develop a way to extract and analyze structural variants, such as CNVs, from the massive and various genetic sequences.

Originally, microarray technology [7,8] and the sequence-based method [9-11] were used to find CNVs in human genetic sequences. In particular, the sequence-based method, a computational approach, compares different genome sequences. This method is known to detect CNVs more accurately than microarray technology, enabling the detection of small or intermediate-sized CNVs. Moreover, the appearance of NGS technology enables rapid and cost-effective sequencing, offering extensive bioinformatic analysis of the generated data and highlighting the importance of computational approaches in finding and analyzing CNVs. The sequence-based method can be divided into two categories: comparing completed assembly sequences and directly using NGS data. The method using NGS data aligns short reads of NGS data onto a completely assembled sequence, a reference sequence, to find CNVs by analyzing the alignment results produced; it is cheaper than the method that compares completely assembled sequences. However, errors in the read data are common, and the probability of errors occurring in the repeat areas of reference sequences is high. It is difficult to find exact locations of CNVs using these data consisting of many errors.

To solve this problem, methods based on paired-end read mapping (PEM), which map paired-end reads made from NGS onto known genome sequences to find CNVs [12]; methods using event-wise testing (EWT) algorithms, which analyze read coverage data in finding CNVs [13]; and methods based on Bayesian statistics [14] have been developed. PEM-based methods, however, make it difficult to find CNVs in areas with complex structural mutation, since these methods rely on technologies for producing paired reads. Methods using EWT algorithms limit the findable CNV size because of their having a fixed window size. Moreover, PEM-based methods, EWT-based methods, and Bayesian statistic-based methods all need high coverage for reads.

Recently, methods based on a sliding window approach, such as CNV-seq [15] and CNV_shape (Hong SK, et al.: Shape-based retrieval of CNV regions in read coverage data, forthcoming), were proposed for detection of even small CNVs at low coverage read data. However, CNV-seq still uses the ratio of the read coverage between control and test sequences, as microarray technology does, causing errors in the decision of CNV types. CNV_shape, which is excellent in the type decision, also has errors in the exact localization of CNVs because it is based on the shape variation of read coverage data. Methods based on the sliding window approach, such as CNV-seq and CNV_shape need to optimize the size of the sliding window according to the coverage and noise levels of target data. However, a larger sliding window size decreases the noise effect and also results in a decrease of the resolution in CNV detection. Therefore, if an initially optimized and fixed sliding window size is used as in both CNV-seq and CNV_shape, small CNVs could be missing due to its limited resolution especially when the coverage level is low and the noise level is high. Therefore, there is an urgent need to develop a method to detect the exact types and locations of CNVs from low coverage read data, since most genome projects need to accurately predict the probability of an individual catching a disease or having genetic disorders by analyzing sequences with fairly low-coverage read data.

This manuscript proposes a new method, CNV_SS, to detect CNVs by using the multi-resolution system of scale-space filtering, enabling the detection of the types and the exact locations of CNVs of all sizes even when the coverage level of read data is low (<5x). The scale-space filtering is a technique that can produce qualitative and hierarchic symbolic descriptions of a signal by transforming it into a continuum of versions, scale-space image, of the original signal convolved with a kernel containing a scale parameter. The scale-space image provides a concise but complete qualitative description, such as local extrema and intervals bounded by dominant points, covering all scales of observation. In this study, the scale-space filtering is evaluated by assuming a Gaussian distribution of read coverage data and the scale-space image is obtained by applying to the read coverage data the Gaussian convolution for various scales according to a given scale parameter. Next, inflection points, zero-crossing points of the second derivatives of the scale-space image are calculated per scale. Then, the types and the exact locations of CNVs are obtained by analyzing the contours of the inflection points of the scale-space image.

## Scale-space filtering

Real-world objects are composed of different structures at different scales. That is, real-world objects may appear in different ways depending on the scale of observation. For example, the concept of a “tree” is appropriate at the scale of meters, while concepts such as leaves and molecules are more appropriate at finer scales. For extracting CNVs of unknown sizes and locations by analyzing read coverage data, there is no way to know a priori what scales are appropriate for describing the structures of CNVs in the read coverage data. Hence, the reasonable approach is to consider descriptions at multiple scales in order to be able to capture the unknown scale variations that may occur. Scale-space filtering, proposed by Witkin, is a method that describes signals qualitatively covering all scales of observation [16]. It is a framework for multi-scale signal representation which handles a signal at different scales and represents it as a one-parameter family of smoothed signals, called scale-space image, parameterized by the size of the smoothing kernel used for suppressing fine-scale structures. The parameter in the family is referred to as the scale parameter. The Gaussian kernel is generally used for smoothing signals, because it is symmetric and readily differentiable, with the standard deviation *σ* as the scale parameter. A signal convolved with the Gaussian kernel satisfies “well-behavedness” criteria, in which the signal is smoothed more as *σ* increases, and eventually approaches the mean value of the signal [17,18].

Let *f*(*t*) be a signal. The scale-space image *f*(*t*,*σ*) of *f*(*t*) is then defined by the convolution of *f*(*t*) and the Gaussian kernel *g*(*t*,*σ*) as follows:

(1) $$f(t,\sigma )=f(t)*g(t,\sigma )=\int_{-\infty }^{+\infty }f(t)\frac{1}{\sigma \sqrt{2\Pi }}e^{-\frac{{\left(t-u\right)}^{2}}{2\sigma^{2}}}\mathrm{du.}$$

Here, zero-crossing points, where the signs of the derivatives of the scale-space image *f*(*t*,*σ*) change, cannot newly appear, but only disappear as *σ* increases, since the scale-space image *f*(*t*,*σ*) gets smoother as *σ* increases. It is particularly useful for the second-order derivative because the zero-crossing points at which $\frac{\delta^{2}f(t,\sigma )}{\delta t^{2}}=0$ represent the inflection points of the scale-space image *f*(*t*,*σ*). Notice that the Gaussian kernel is the only one guaranteed to satisfy this property [19].

**Figure 1:**
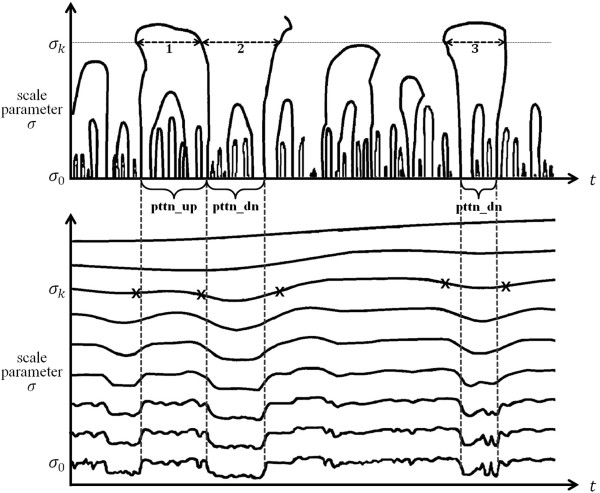
**A typical scale-space image and the finger print map.** A typical scale-space image with increasing *σ* (on the bottom) and the contours of the zero-crossing points of the second derivatives of the scale-space image (on the top) (adopted from [16]). The vertical dotted lines show the inflection points of the scale-space image at the lowest *σ*(=*σ*_0_) and the horizontal arrowed lines, intervals at a *σ*(=*σ*_*k*_) between two neighboring inflection points, along with the corresponding points marked by “x” on the scale-space image at *σ*(=*σ*_*k*_).

Figure 1 represents a typical scale-space image with increasing *σ* (on the bottom) and the contours of the zero-crossing points of the second derivatives of the scale-space image (on the top) [16]. Here, the vertical dotted lines show the inflection points of the scale-space image at the lowest *σ*(=*σ*_0_) and the horizontal arrowed lines, intervals at a *σ*(=*σ*_*k*_) between two neighboring inflection points along with the corresponding points marked by “x” on the scale-space image at *σ*(=*σ*_*k*_). Each interval represents a pattern of the signal. Specifically, the interval numbered 1 represents the pattern of concave down (pttn_up) and the intervals numbered 2 and 3 represent the pattern of concave up (pttn_dn) in Figure 1. The collection of the contours is called the finger print map of the scale-space image. The contours open downward, and closed upward due to the characteristic of the zero-crossing points of the second derivatives of the scale-space image. Each contour represents one of the two patterns, concave up and down of a signal, which is specified by two neighboring inflection points. The pattern of a signal is more easily detected from intervals for larger *σ* since larger *σ* is less prone to noise. An interval at a scale is smoothed out for some large *σ* as shown in Figure 1. Also, an interval at a scale may enclose intervals at lower scales, which means the enclosed intervals of lower scales may be treated as small signals (noises) within the interval at a scale. However, the positions of inflection points may be shifted outward for large *σ* because of the convolutions, expanding the intervals for large *σ*. Therefore, once a pattern of a signal is defined from an interval at large *σ*, the accurate location of the pattern should be found by tracing the convergence of the interval through the contours along the scale parameter *σ* downward to the initial value.

CNV is one of alterations of the DNA of a genome that results in the cell having an abnormal number of copies of one or more sections of the DNA. CNV regions on read coverage data can be defined as regions where the levels of the coverage vary greatly between different regions. Therefore, in terms of scale-space filtering, CNV regions can be regarded as intervals bounded by dominant points where the read coverage data vary greatly between different regions. In other words, CNV regions can be regarded as intervals between two neighboring inflection points, one from positive curvature to negative curvature and the other from negative curvature to positive curvature, vice versa. In CNV_SS, scale-space filtering evaluates scale-space image by applying the read coverage data the Gaussian convolution for various scales according to a given scale parameter. The scale-space image represents noise-filtered read coverage data with variable sliding window size corresponding to the scale parameter for every scale. CNV_SS selectively chooses the scale for CNV detection depending on the coverage level as well as the size of CNVs, that is to have adaptable window size for various noise levels as well as various CNV sizes and then localizes the exact position of the CNV at the lowest scale.

## Methods

**Figure 2:**
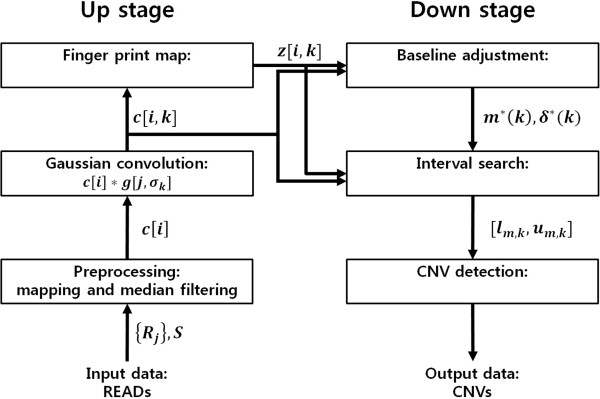
**The overall processes of CNV_SS.** CNV_SS proceeds in two stages: up and down stages. The up stage includes preprocessing, Gaussian convolution, and finger print mapping. The down stage includes baseline adjustment, interval search, and CNV detection.

CNV_SS proceeds in two stages: up and down stages. Figure 2 describes the overall processes. The up stage includes preprocessing, Gaussian convolution, and finger print mapping. The down stage includes baseline adjustment, interval search, and CNV detection.

First, in the up stage, read coverage data are generated by mapping reads of input data to a reference genome. Then, they are decomposed into *l* layers by Gaussian convolution with increasing *σ*. Next, the zero-crossing points of the second-order derivatives of the decomposed data are evaluated per layer. Finally, a finger print map is obtained from the zero-crossing points.

In the down stage, the baselines of each layer are calculated using the mean and the standard deviation of the read coverage data for each layer with decreasing *σ*. Intervals are also searched by using the baselines through the finger print map with decreasing *σ*. Here, an interval is a region of the input sequence where a CNV gain or loss is detected. More than one interval is not permitted in a region of the sequence. Therefore, once an interval is obtained at a layer, the exact position of the detected CNV is decided by localizing the positions where the start and the end points of the interval converge at the lowest layer; no more interval searching at the corresponding region is necessary at the sub-layers.

### Preprocessing

**Figure 3:**
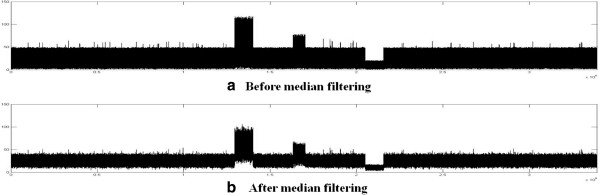
**An example of virtual read coverage data.** The read coverage data are median filtered to reduce the noise errors before proceeding to the Gaussian convolution; (**a**) and (**b**) show virtual read coverage data before and after the median filtering, respectively.

Read coverage data are generated by aligning reads of input data to a given reference sequence, and then they are filtered by a median filter. The median filter is an effective method that can suppress isolated noises without blurring sharp edges of a signal which is affected by the size of the sliding window. In other words, the size of the sliding widow in median filtering should be chosen not to blur sharp edges of the signal. The read coverage data *c*[*i*]=*c*_1_*c*_2_…*c*_*n*_ consist of a series of the number *c*_*i*_ of reads aligned to the genome position *i* (1 ≤ i ≤ n). The read coverage data *c*[*i*] are median filtered to reduce the noise errors before proceeding to the Gaussian convolution. The median filtering is accomplished by replacing each entry *c*_*i*_ with the median of entries, *c*_*i*−*w*/2_…*c*_*i*_…*c*_*i*+*w*/2_ in a sliding window with size *w*+1. The value for *w* was set to 150 which was determined as around 15% of the smallest CNV size by a heuristic evaluation in order to keep CNV signals while suppressing isolated noises in the read coverage data. Figures 3(a) and 3(b) show virtual read coverage data before and after the median filtering, respectively. It can be seen that the large spikes are reduced and the data become less noisy after filtering.

### Gaussian convolution

The read coverage data *c*[*i*]=*c*_1_*c*_2_…*c*_*n*_ are decomposed into *l* layers by Gaussian convolution with increasing *σ* as in the following equation:

(2) $$c[i,k]=c[i]*g[j,\sigma_{k}]=\overset{m}{\underset{j=-m}{\sum }}c[i-j]\frac{1}{\sigma_{k}\sqrt{2\Pi }}e^{-\frac{j^{2}}{2\sigma_{k}^{2}}},$$

where *c*[*i*,*k*] is called the scale-space image of *c*[*i*]=*c*_1_*c*_2_…*c*_*n*_, *k* (0≤*k*≤*l*−1) represents the index of the layer of the scale-space image, *σ*_*k*_ is the value of the scale parameter at layer *k*, and *m* is the window size of the Gaussian kernel *g*[*j*,*σ*_*k*_], which is set to *m*=3*σ*_*k*_. The scale parameter *σ*_*k*_ is the standard deviation of the Gaussian kernel *g*[*j*,*σ*_*k*_], and is set to *σ*_*k*_=10^3^×(1.1)^*k*^ considering the range of detectable CNV size and time complexity. The range of the scale parameter *σ*_*k*_ determines the range of CNV size detectable in this method. The ratio of two adjacent scales determines both the time complexity and the resolution in CNV detection. The smaller ratio of two adjacent scales, the better in resolution of CNV detection but the worse in the time complexity. Therefore, trading off between the resolution and the time complexity can be controlled by the ratio of two adjacent scales. The value of *σ*_*k*_ increases exponentially as *k* increases.

**Figure 4:**
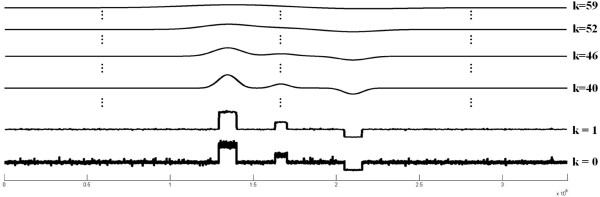
**An example of a scale-space image.** The scale-space image obtained by applying the read coverage data of Figure 3(b) the Gaussian convolution for various scale parameters of *σ*_*k*_, *k* = 0, 1, 40, 46, 52, and 59.

The computational complexity to get a scale-space image *c*[*i*,*k*] of *c*[*i*] is *O*((1.1)^*l*^*n*). It is well known that the convolution in the time domain is the same as the product in the frequency domain. Therefore, we obtain the scale-space image *c*[*i*,*k*] of *c*[*i*] by applying discrete Fourier transform in frequency domain to reduce the computational complexity. Let *C*[*w*] and $G[w,k]=e^{-w^{2}\sigma_{k}^{2}/2}$ be the discrete Fourier transform of *c*[*i*], and *g*[*j*,*σ*_*k*_], respectively. The scale-space image is then obtained by

(3) $$c[i,k]=F^{-1}\{G[w,k]C[w]\},$$

where *F*^−1^ is the inverse discrete Fourier transform operator. The computational complexity to get a scale-space image *c*[*i*,*k*] of *c*[*i*] by discrete Fourier and discrete inverse Fourier transform is *O*(*nlog*_2_*n*) if the fast Fourier transform algorithm is used. When the number of the layers of the scale-space image is large, the computing time can be greatly reduced in the frequency domain. Specifically for *n*=10^6^, *l*=60, the computational complexity can be reduced about 1,000 times. Figure 4 shows the scale-space image obtained by using the read coverage data of Figure 3(b) representatively for *k* = 0, 1, 40, 46, 52, and 59.

### Finger print mapping

The zero-crossing points of the second-order derivatives of the scale-space image *c*[*i*,*k*] are searched for each layer *k*(0 ≤ *k* ≤ *l* − 1). Here, the second derivative *c*^′′^[*i*,*k*] of *c*[*i*,*k*] is approximated by the second-order difference, that is, *c*^′′^[*i*,*k*] ≈ *c*[ *i* + 1, *k*] − 2*c*[ *i*,*k*] +*c*[ *i* − 1,*k*]. A zero-crossing signal *z*[ *i*,*k*] is defined as follows:

(4) $$z[i,k]=\left\{\begin{array}{l} +1,\;c^{\prime \prime }[i+1,k]>0\;\text{and}\;c^{\prime \prime }[i-1,k]<0\\ -1,\;c^{\prime \prime }[i+1,k]<0\;\text{and}\;c^{\prime \prime }[i-1,k]>0,\\ \;\;0,\;\text{else} \end{array}\right)$$

where the condition *c*^′′^[ *i* + 1,*k*] > 0 and *c*^′′^[ *i* − 1,*k*] < 0 represent the zero crossing point *i* at which *c*^′′^[ *i*,*k*] crosses zero from minus to plus, and the conditions *c*^′′^[ *i*+1,*k*] < 0 and *c*^′′^[ *i*− 1,*k*] > 0, from plus to minus.

**Figure 5:**
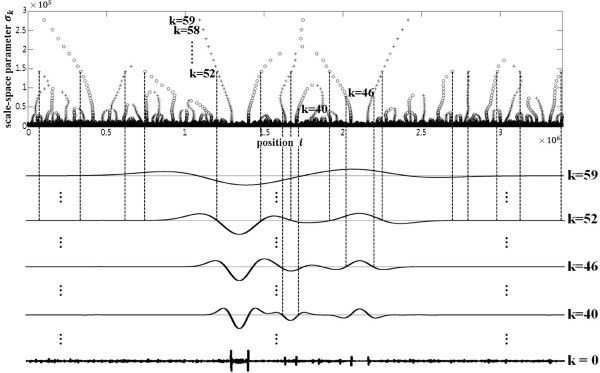
**A plot of the zero-crossing signal with the second derivatives of the scale-space image of Figure **4**.** A plot of zero-crossing signal *z*[*i*,*k*], also called a finger print map on the top, along with the second derivatives on the bottom, representatively for *k*=40, 46, 52, and 59, of the scale-space image described in Figure 4, where the zero-crossing points from minus to plus (*z*[*i*,*k*] = +1) are denoted by ‘o’, and from plus to minus (*z*[*i*,*k*]= −1) by ‘+’.

Figure 5 shows a plot of zero-crossing signal *z*[ *i*,*k*], also called a finger print map on the top, along with the second derivatives on the bottom, representatively for *k* = 40, 46, 52, and 59, of the scale-space image described in Figure 4, where the x-axis represents the position *i* on the reference sequence and the y-axis, the value of the scale parameter *σ*_*k*_ for 0 ≤ *k* ≤ *l* − 1. In the figure, the values +1 and −1 of *z*[*i*,*k*] are represented by markers ‘o’ and ‘+’, respectively. The values 0 of *z*[ *i*,*k*] are not marked in the figure. The vertical dotted lines indicate the zero-crossing points. All the zero-crossing points for *k* = 52 are indicated by the vertical dotted lines but some of them for *k* = 40 and 46.

### Baseline detection

At the down stage, three baselines, *m*^∗^(*k*), *m*^∗^(*k*)+*dδ*^∗^(*k*), and *m*^∗^(*k*)−*dδ*^∗^(*k*) are calculated using the effective mean *m*^∗^(*k*) and the effective standard deviation *δ*^∗^(*k*) of the scale-space image *c*[*i*,*k*] for each of the layers that have more than two non-zero elements in zero-crossing signal *z*[*i*,*k*], where *d* is a parameter for the baseline adjustment. Here, we set the value of *d* to 3. The effective mean *m*^∗^(*k*) and the effective standard deviation *δ*^∗^(*k*) at layer *k* are evaluated by the average and the standard deviation of *c*[*i*,*k*], respectively, excluding the points whose values are out of the normal range. The normal range is set at *m*(*k*)±2*δ*(*k*), where $m(k)=\underset{i}{\sum }c[i,k]/\underset{i}{\sum }$ and $\delta (k)=\sqrt{\underset{i}{\sum }{\left(c[i,k]-m[k]\right)}^{2}/\underset{i}{\sum }}$ are the mean and the standard deviation of *c*[ *i*,*k*], respectively.

### Interval search

Intervals are searched from the zero-crossing signal *z*[*i*,*k*] using the baselines *m*^∗^(*k*)±3*δ*^∗^(*k*) for each of the layers that have more than two non-zero elements in zero-crossing signal *z*[*i*,*k*]. The *m*th interval [*l*_*m*,*k*_,*u*_*m*,*k*_] at layer *k* is defined as a closed interval {*i*|*l*_*m*,*k*_≤*i*≤*u*_*m*,*k*_} in the position index *i* of the zero-crossing signal *z*[*i*,*k*], that is a set of the position indices of *z*[*i*,*k*] between *l*_*m*,*k*_ and *u*_*m*,*k*_ inclusive, satisfying the following three conditions to be a putative CNV region. First, interval [*l*_*m*,*k*_,*u*_*m*,*k*_] does not include position indices corresponding to all the regions of CNVs already declared at layers above the layer *k*. Second, *z*[ *l*_*m*,*k*_, *k*] • *z*[ *u*_*m*,*k*_, *k*] < 0 and *z*[ *i*,*k*] = 0 for all the position indices between *l*_*m*,*k*_ and *u*_*m*,*k*_. Third, the average $\overset{u_{m,k}}{\underset{i=l_{m,k}}{\sum }}c[i,k]/(u_{m,k}-l_{m,k}+1)$ of the scale-space image on the position indices between *l*_*m*,*k*_ and *u*_*m*,*k*_ inclusive is beyond the given baselines, *m*^∗^(*k*)−3*δ*^∗^(*k*) or *m*^∗^(*k*)+3*δ*^∗^(*k*).

Once we have the *m*th interval [*l*_*m*,*k*_,*u*_*m*,*k*_] as a putative CNV region, then we trace the zero-crossing signal *z*[*i*,*k*] from the positions *l*_*m*,*k*_ and *u*_*m*,*k*_ at layer *k* until we get the corresponding positions $l_{m,k}^{\prime }$ and $u_{m,k}^{\prime }$, respectively bounded at layer *k*=0, where the closed interval $\left[l_{m,k}^{\prime },u_{m,k}^{\prime }]=\{i|l_{m,k}^{\prime }\leq i\leq u_{m,k}^{\prime }\right\}$ is to be declared as a CNV. In other words, the interval $\left[l_{m,k}^{\prime },u_{m,k}^{\prime }\right]$ is a precisely fine tuned CNV region corresponding to the interval [*l*_*m*,*k*_,*u*_*m*,*k*_], a putative CNV detected at layer *k*. CNV search is proceeded from the top layer to the bottom layer, layer by layer. Therefore, for searching intervals at layer *k*, the sum of sets, $\overset{k_{\text{max}}}{\underset{s=k+1}{⋃}}\overset{m_{\text{max,s}}}{\underset{m=1}{⋃}}[l_{m,s}^{\prime },u_{m,s}^{\prime }]$ corresponding to all the regions of CNVs already declared at the upper layers from *k*+1 to *k*_*max*_ should be excluded, where *m*_*max,s*_ is the total number of CNVs detected at layer *s*. In other words, all the sum of intervals, $\overset{m_{\text{max,k}}}{\underset{m=1}{⋃}}[l_{m,k},u_{m,k}]$ searched at layer *k* should not be overlapped with the sum of intervals, $\overset{k_{\text{max}}}{\underset{s=k+1}{⋃}}\overset{m_{\text{max,s}}}{\underset{m=1}{⋃}}[l_{m,s}^{\prime },u_{m,s}^{\prime }]$ already declared as CNVs at the upper layers, which is the first condition. The second condition is that if *l*_*m*,*k*_ is a zero crossing point from minus to plus then *u*_*m*,*k*_ should be from plus to minus, vice versa as well as there are no other zero crossing points between *l*_*m*,*k*_ and *u*_*m*,*k*_. The meaning of the second condition is that the values of the scale space image *c*[*i*,*k*] between *l*_*m*,*k*_ and *u*_*m*,*k*_, inclusive have significantly different values from others of *c*[*i*,*k*] to be declared as a CNV. The third condition is for the definition of baselines *m*^∗^(*k*), *m*^∗^(*k*)±3*δ*^∗^(*k*), the guidelines for determining whether the values of the scale space image *c*[*i*,*k*] between *l*_*m*,*k*_ and *u*_*m*,*k*_, inclusive have significantly different from others of *c*[*i*,*k*] for 1≤*i*≤*n*, where *m*^∗^(*k*) and *δ*^∗^(*k*) are the effective mean and the effective standard deviation of *c*[*i*,*k*] for 1≤*i*≤*n* at layer *k*, respectively.

**Figure 6:**
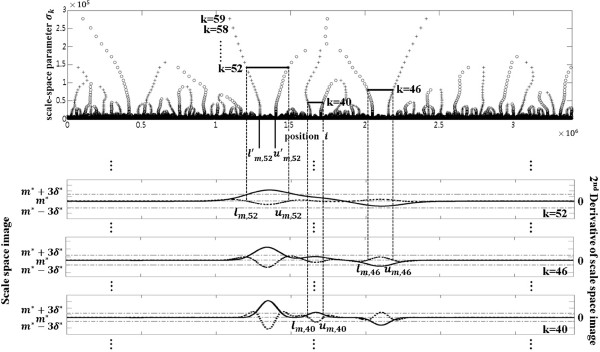
**Finger print map of the scale-space image of Figure**4**.** Finger print map of the scale-space image of Figure 4 with intervals at layers *k*=40, 46, and 52, along with the graphs of the scale-space images (solid curve) and the second derivatives (dotted curve) for *k*=40, 46, and 52 from the bottom, respectively, where *m*^∗^ + 3*δ*^∗^, *m*^∗^, and *m*^∗^ − 3*δ*^∗^ depicted by horizontal dotted lines in each of the graphs represented the corresponding baselines.

Figure 6 shows the finger print map of the scale-space image of Figure 4 with intervals at layers *k*=40, 46, and 52 along with the graphs of the scale-space images (solid curve) and the second derivatives (dotted curve) for *k*=40, 46, and 52 from the bottom, respectively. Here, the vertical dotted lines represent the intervals [*l*_*m*,*k*_,*u*_*m*,*k*_] and the horizontal dotted lines represent the baselines *m*^∗^(*k*) and *m*^∗^(*k*)±3*δ*^∗^(*k*) for each of the layers *k*=40, 46, and 52. As shown in Figure 6, the intervals presented satisfy the three conditions described above.

### CNV detection

The type and the localization of a CNV are determined by using the results of interval search. An interval [*l*_*m*,*k*_,*u*_*m*,*k*_] identifies the region where a statistically significant variation occurs on the input sequence and a CNV gain or loss is to be detected. That is, a CNV gain or loss is identified if the average $\overset{u_{m,k}}{\underset{i=l_{m,k}}{\sum }}c[i,k]/(u_{m,k}-l_{m,k}+1)$ of scale-space image in the interval is above *m*^∗^(*k*)+3*δ*^∗^(*k*) or below *m*^∗^(*k*)−3*δ*^∗^(*k*), respectively. Then the localization of a CNV is defined by tracing to the corresponding region $\left[l_{m,k}^{\prime },u_{m,k}^{\prime }\right]$ as the layer *k* converges to zero. Figure 6 shows a CNV calling as a gain by the vertical solid lines below the finger print map representing the corresponding region $\left[l_{m,k}^{\prime },u_{m,k}^{\prime }\right]$ of the interval [*l*_*m*,*k*_,*u*_*m*,*k*_] at *k*=52 where the average of the scale-space image *c*[*i*,*k*] in the interval [*l*_*m*,*k*_,*u*_*m*,*k*_] is above *m*^∗^(*k*)+3*δ*^∗^(*k*).

### Materials

A simulation data generator (SDG) was developed to generate simulated data. It generates a reference sequence and a test sequence, which contain CNVs of various sizes and types, as well as single nucleotide polymorphisms (SNPs) and short indels. The SDG starts with a given DNA sequence both as a reference sequence and as a test sequence. It then copies some of the CNVs of the sequence referring to the CNV database of the Database of Genomic Variants (DGV; http://projects.tcag.ca/variation) and substitutes them in random positions of the reference sequence or the test sequence so that the test sequence has CNV gains or losses that differ in size and location compared with the reference sequence. An indel is constructed by inserting or deleting a short sequence at a random position of the reference or the test sequence. For SNPs, the SDG replaces the nucleotides at random positions of the test sequence so that each of the replaced positions in the test sequence has a different nucleotide from that in the reference sequence. Once a reference sequence and a test sequence are generated, reads of the test sequence are generated by simulating the shotgun sequencing process of the Solexa machine.

A total of 80 simulated data were generated by SDG, in which NCBI Build 36.3 chromosome (chr) 8 genomic contig NT_077531.3 was used as the starting sequence and the corresponding information of the 17 potential CNV regions (total length of 318,750 bp, average length of 18,750 bp, minimum length of 1,024 bp, and maximum length of 70,613 bp) referring to DGV were used. Specifically, 10 of the 17 potential CNV regions were randomly selected and inserted in random positions of the reference sequence or the test sequence. The test sequences were treated to have a given sequencing error rate (E%). Then, paired-end reads of length 36 bp were randomly extracted at a given read coverage (C) from each of the 80 simulated test sequences.

Read data downloaded from the site of the 1000 Genome Project (http://www.1000genomes.org) were used for the experiment with real human data. The downloaded data were paired-end reads of six hapmap samples: NA10851 (5.6x), NA18511 (1.8x), NA18570 (2.2x), NA18576 (1.8x), NA18592 (2.3x), and NA18944 (2.3x), which were generated by the Solexa GA machine.

The performance of CNV_SS was assessed by comparing the detected CNV regions with those reported to DGV on each individual, in which we used the CNV database of DGV updated on November 2, 2010. CNV_SS was also compared with three other CNV detection methods: CNV_shape (Hong SK, et al.: Shape-based retrieval of CNV regions in read coverage data, forthcoming), CNV-seq [15], and modified CNV-seq (Hong SK, et al.: Shape-based retrieval of CNV regions in read coverage data, forthcoming) with optimized parameters for each method.

SOAP2 (Short Oligonucleotide Alignment Program) [20] was used for the alignment of the read data, and a random match method, one of various alignment algorithms that SOAP2 supports, was used with e = 2 mismatch criteria as a tolerable limit with regard to noise, such as sequence errors. The experiments were carried out in the platform of Windows 7 and CentOS 5.5 on Intel Core i7 2.8GHz CPU, 8GB main memory, and 2TB hard drive. The programming language used for the development of CNV_SS was MATLAB.

## Results and discussion

### Experiments with simulated data

The first experiment was carried out to assess the performance of CNV_SS for various read coverage levels: 0.1x, 0.5x, 1x, 2x, 3x, 6x, and 10x. Here, the sequencing error rate E=2% was considered according to a typical error rate existing in real data generated by shotgun sequencing technology, even though the sequence error rate keeps improving due to the advancement of the technology. Performance was assessed by estimating the false negative rate (FNR) and the false positive rate (FPR) on the basis of the size of detected CNV regions. More than 20 experiments for each read coverage level were accomplished with different simulated data, the results from which were averaged for the assessment. The performance of the CNV_SS was then compared with those of CNV_shape and CNV-seq.

**Figure 7:**
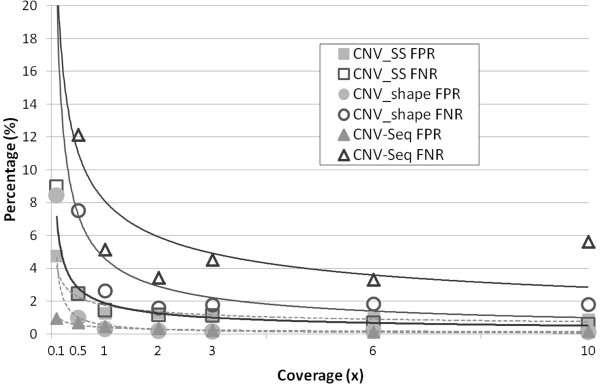
**Performance vs coverage level.** Comparison of the performances of three algorithms for simulated data of coverage level C in the range of 0.1x to 10x. The sequence error rate E was set to 2%. Performance was assessed by estimating the false negative rate (FNR) and the false positive rate (FPR) on the basis of the size of detected CNV regions.

Figure 7 shows the results of the experiments for various read coverage levels at the sequencing error rate E=2%. As shown in Figure 7, most algorithms are fairly good at the detection of CNVs at high coverage levels. However, CNV_SS shows better performance in FNR than other methods at low coverage levels. Overall FNRs and FPRs of CNV_SS were in the range of 0.58% to 8.96% and 0.87% to 4.76%, with medians of 1.14% and 1.271%, respectively. The FNRs and FPRs decrease as the read coverage level increases. The decreasing rates also get lower as the read coverage level increases, resulting in the saturation of FPRs around at the read coverage level of 2x or 3x. CNV_SS has fairly good FNR (2.44%) and FPR (2.43%) even at a very low level of read coverage C=0.5x.

Compared with CNV_shape and CNV-seq, CNV_SS gave a little increased FPRs. This is due to the fact that CNV_SS searches CNVs at every layer of the scale-space image from the top to the bottom not to exclude small (around 1 Kbp) CNVs, inevitably resulting in calling small noise signals as CNVs as well. To exclude noise signals in CNV detection, our future work considers an algorithm for selectively adjusting the range of layers of the scale-space image according to the properties of the input data set in CNV detection, which will decrease FPRs even at very low coverage levels.

**Figure 8:**
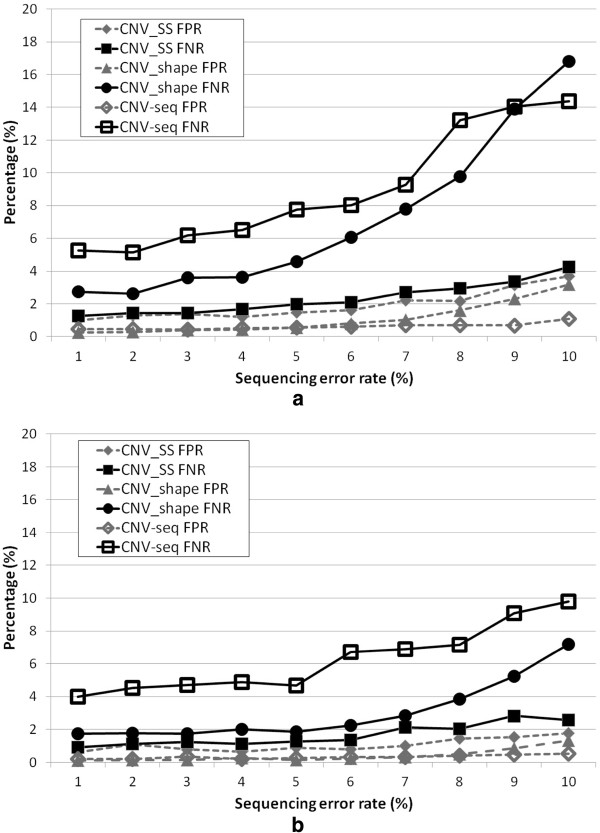
**Performance vs sequencing error rate.** Comparison of the performances of three algorithms for simulated data of sequence error rate E in the range of 1% to 10%. Performance was assessed by estimating the false negative rate (FNR) and the false positive rate (FPR) on the basis of the size of detected CNV regions; (**a**) and (**b**) show the results for coverage levels of C=1x and C=3x, respectively.

The second experiment was carried out to assess CNV_SS for various sequencing error rates, 1% through 10%. Figures 8(a) and 8(b) show the FNRs and FPRs as the sequencing error rate increases when the read coverage levels are 1x and 3x, respectively. The FNRs and FPRs increase as the sequencing error rate increases. As can be seen in Figure 8(a), the FNRs both for CNV-seq and the CNV_shape increase rapidly as the sequencing error rate increases when the read coverage level is low. While CNV_SS has fairly good FNR (4.25%) and FPR (3.71%) even when the sequencing error rate E is high (10%) and the level of read coverage C is low (1x). The overall FNRs and FPRs for CNV_SS were in the range of 1.25% to 4.25% and 0.98% to 3.71%, respectively, at the read coverage level C=1x, and in the range of 0.92% to 2.56% and 0.63% to 1.78%, respectively, at the read coverage level C=3x. The result suggests that CNV_SS can be very robust in error-prone environments at a moderate level of the read coverage.

**Figure 9:**
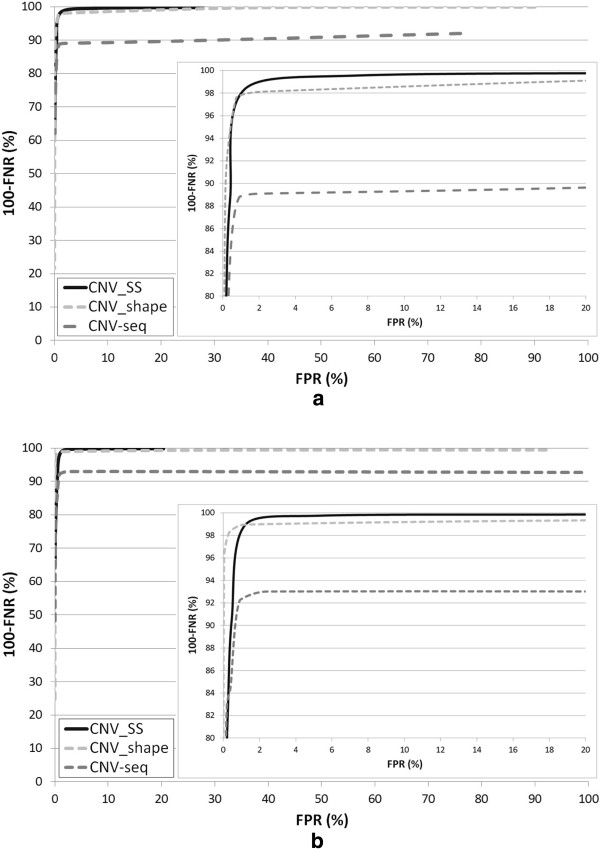
**ROC curves for three algorithms at different coverage levels and error rates.** The ROC curves are generated by measuring false positive and false negative rates on simulated data at different threshold levels; (**a**) and (**b**) show the ROC curves for the cases of (C=1x, E=4%) and (C=3x, E=2%) data sets, respectively.

The third experiment was carried out to assess the performance of CNV_SS in conjunction with two other methods as the threshold for determining a CNV is varied. We calculated the receiver operation characteristic (ROC) curves of CNV_SS using two data sets. These ROC curves, together with the ROC curves of CNV-seq and CNV_shape based on the same data sets, were plotted in Figure 9. Figures 9(a) and 9(b) show the ROC curves for the cases of (C=1x, E=4%) and (C=3x, E=2%) data sets, respectively. These curves show that CNV_SS and CNV_shape are more sensitive than CNV-seq. As CNV_SS detects larger CNVs at higher scale and smaller CNVs at lower scale, the sensitivity can be increased compared to the conventional methods using a fixed window size. For the case of lower coverage level and higher error, CNV_SS gave better performance results than other methods.

### Experiments with hapmap samples

Paired-end reads of six hapmap samples, NA10851 (5.6x), NA18511 (1.8x), NA18570 (2.2x), NA18576 (1.8x), NA18592 (2.3x), and NA18944 (2.3x), were used for the experiments with real human data. FPR and FNR were evaluated on the basis of the CNV database of the DGV and then compared with those of CNV_shape, the conventional CNV-seq, and the modified CNV-seq.

In the first experiment, paired-end reads from the human leukocyte antigen (HLA) region of chr. 6 of NA18511 (1.8x) and NA10851 (5.6x) were used. The HLA region, which resides on the short arm of human chr. 6 and is 3.408 Mbp long, is known to have around 200 genes related to the human immune system and several potential CNV regions involving disease-specific genes [21].

**Figure 10:**
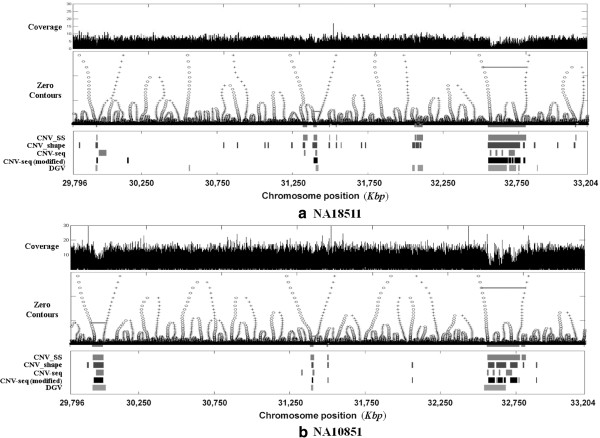
**Detected CNV regions.** CNV regions detected by four methods in the HLA regions of (**a**) NA18511 and (**b**) NA10852. The top panels of Figures 10(a) and 10(b) show the graphs of the read coverage data along with the position of chr. 6 of NA18511 and NA10851, respectively. The middle panels of Figures 10(a) and 10(b) display the finger print maps of the read coverage data of NA18511 and NA10851, respectively. The bottom panels of Figures 10(a) and 10(b) show the CNV regions detected by each of CNV_SS, CNV_shape, CNV-seq, and modified CNV-seq on NA18511 and NA10851, respectively; the same panels show the CNV regions reported in the DGV.

Figure 10 shows the results of the experiment on the HLA regions of NA18511 and NA10851. The top panels of Figures 10(a) and 10(b) show the graphs of the read coverage data along the position of chr. 6 of NA18511 and NA10851, respectively. The middle panels of Figures 10(a) and 10(b) display the finger print maps of the read coverage data of NA18511 and NA10851, respectively; the x-axis is the position of chr. 6, and the y-axis represents the values of the scale parameter *σ*. The bottom panels of Figures 10(a) and 10(b) show the regions of CNVs detected by CNV_SS on NA18511 and NA10851, respectively; the same panels show the regions of CNVs detected by each of CNV_shape, CNV-seq, and modified CNV-seq for comparison, and the CNV regions reported in the DGV. A total of 10 CNVs (minimum size, maximum size, and total sum of the regions are 1,124 bp, 117,689 bp, and 239,961 bp, respectively) for HLA of chr. 6 on NA18511 and 3 CNVs (minimum size, maximum size, and total sum of the regions are 10,175 bp, 142,219 bp, and 236,332 bp, respectively) on NA10851 are reported in the DGV.

As shown in the middle and the bottom panels of Figure 10, CNV_SS accurately detects the CNV types, gain or loss, for both NA18511 and NA10851, which is considered because of the typical characteristics of the scale-space filtering. However, CNV_shape may incorrectly detect the types of small CNV regions when the noise distribution is irregular in low-coverage sequencing data, because the method is based on the variations in the shape of the read coverage data. Furthermore, CNV-seq cannot verify the types of detected CNVs because the method is based on the coverage ratio of test to control samples. As shown in the bottom panels of Figures 10(a) and 10(b), CNV-seq failed to detect as CNVs a region of 32,578,489 bp through 32,608,297 bp on chr. 6 of NA18511 and a region of 32,578,489 bp through 32,608,297 bp on chr. 6 of NA10851, which are both reported as CNV loss regions in the CNV database of the DGV. Furthermore, the region 31,409,353 bp through 31,419,289 bp on chr. 6 was detected as a CNV loss by CNV_SS, while it was estimated to be a CNV gain on NA18511 and, at the same time, a CNV loss on NA10851 by CNV-seq, as shown from the top panels of Figures 10(a) and 10(b).

Table 1 summarizes a quantitative analysis of the results of the experiments on the HLA regions of NA18511 and NA10851, in which the performance of CNV_SS was compared with that of CNV_shape, the conventional CNV-seq, and the modified CNV-seq through the FPRs and the FNRs. The columns ‘Min’ and ‘Max’ represent the smallest and the largest sizes of the CNVs detected by each method, and overlap with those reported in the DGV as well. The fraction of the overlapping is also given in each of the columns ‘Min’ and ‘Max’ by parentheses. The column ‘Gain’ (‘Loss’) represents the total sum of the sizes of CNV gains (losses) detected by each method.

**Table 1:** Summary of performance in HLA regions of NA18511 (1.8x) and NA10851 (5.6x)

**Method**	**NA18511**						**NA10851**					
	**Detected region (Kbp)**				**FPR(%)**	**FNR(%)**	**Detected region (Kbp)**				**FPR(%)**	**FNR(%)**
	**Min**	**Max**	**Gain**	**loss**			**Min**	**Max**	**Gain**	**Loss**		
CNV_SS	5.9(100)	117(100)	21	307	4.03	16.31	10(100)	142(100)	0	378	5.13	8.99
CNV_shape	4.4(85)	117(100)	14	217	1.98	35.10	10(47.7)	142(44.6)	3	134	2.73	26.40
CNV-seq	16(51.9)	117(22.5)	77	46	0.04	72.93	10(57.2)	142(18.6)	46	77	1.57	68.47
CNV-seq (modified)	5.9(100)	117(85.1)	13	179	0.04	43.97	10(53.7)	142(55.7)	0	203	1.78	39.01

As shown in Table 1, the smallest CNV detected by CNV_SS has a 100% overlap with the smallest 10 Kbp CNV in DGV on NA10851, while the smallest ones detected by CNV_shape, CNV-seq, and modified CNV-seq have 47.7%, 57.2%, and 53.7% overlaps with the smallest 10 Kbp CNV in DGV on NA10851, respectively. The largest CNV detected by CNV_SS has a 100% overlap with the largest 142 Kbp CNV in DGV on NA10851, while the largest ones detected by CNV_shape, CNV-seq, and modified CNV-seq have 44.6%, 18.6%, and 55.7% overlaps with the largest 142 Kbp CNV in DGV on NA10851, respectively.

For NA18511, any method listed in Table 1 does not have a CNV call on the smallest 1.12 Kbp CNV in DGV, which is regarded due to the low level of read coverage data of NA18511. The smallest CNV detected by CNV_SS has a 100% overlap with the 5.9 Kbp CNV in DGV on NA18511, while the smallest ones detected by CNV_shape, CNV-seq, and modified CNV-seq have 85.0%, 51.9%, and 100% overlaps with 4.4 Kbp, 16.0 Kbp, and 5.9 Kbp CNVs in DGV on NA18511, respectively. The largest CNV detected by CNV_SS has a 100% overlap with the largest 117 Kbp CNV in DGV on NA18511, while the largest ones detected by CNV_shape, CNV-seq, and modified CNV-seq have 100%, 22.5%, and 85.1% overlaps with the largest 117 Kbp CNV in DGV on NA18511, respectively.

FNRs of 16.31% and 8.99% were derived for NA18511 and NA10851 in CNV_SS. In contrast, CNV_shape yielded FNRs of 35.10% and 26.40%. CNV-seq and the modified CNV-seq yielded FNRs of 72.93% and 43.97% for NA18511 and 68.47% and 39.01% for NA10851, respectively.

In the second experiment, paired-end reads from the whole region of human chr. 6 of NA18511 and NA10851 were used. Human chr. 6 is 170.899 Mbp long, and a total of 75 CNVs (minimum size, maximum size, and total sum of the regions are 1,045 bp, 117,686 bp, and 965,011 bp, respectively) and 54 CNVs (minimum size, maximum size, and total sum of the regions are 1,057 bp, 142,219 bp, and 606,194 bp, respectively) are reported in the CNV database of the DGV for chr. 6 on NA18511 and NA10851, respectively.

Table 2 describes a quantitative analysis of the results of the experiment on the whole region of human chr. 6 of NA18511 and NA10851, in which the performance of the CNV_SS was compared with that of CNV_shape, the conventional CNV-seq, and the modified CNV-seq through FPRs and FNRs. The comparison of the performance result of each method in Table 1 with that in Table 2 reveals that the FNRs are a little increased in the whole region of chr. 6. This is likely to be due to the special characteristics of the HLA region where many CNV regions, including large-scale ones, are well studied and reported; although the HLA region occupies only around 2% of the whole region of chr. 6, 38% and 24% among the CNV regions reported on chr. 6 are within the HLA regions on NA18511 and NA10851, respectively.

**Table 2:** Comparative summary of performance in the whole regions of human chromosome 6 of NA18511 and NA10851

**Method**	**NA18511**						**NA10851**					
	**Detected region (Kbp)**				**FPR(%)**	**FNR(%)**	**Detected region (Kbp)**				**FPR(%)**	**FNR(%)**
	**Min**	**Max**	**Gain**	**loss**			**Min**	**Max**	**Gain**	**Loss**		
CNV_SS	1.8(100)	117(100)	473	438	2.12	41.08	1.3(100)	142(100)	361	1690	2.77	17.31
CNV_shape	1.1(85)	117(98.8)	13730	2515	9.39	45.32	1.1(100)	142(90.5)	1020	931	0.92	24.94
CNV-seq	4.1(100)	117(8.8)	288	63	0.13	86.29	6.2(100)	142(7.2)	63	288	0.04	75.17
CNV-seq (modified)	4.1(100)	117(100)	602	730	0.56	57.68	1.3(100)	142(55.7)	538	757	0.54	40.00

The sizes of CNVs detected by CNV_SS on NA18511 and NA10851 are in the range of 1.8 Kbp to 117 Kbp and 1.3 Kbp to 142 Kbp, respectively. These results confirm that CNV_SS is superior to CNV_shape, the conventional CNV-seq, and the modified CNV-seq in terms of detecting CNVs of various sizes. The results also show that small CNVs can be accurately detected from low-coverage data. We can deduce therefore that CNV_SS is very effective at reducing the noise inherent in the read coverage data and in detecting CNVs of various sizes and types.

In the third experiment, paired-end reads from the whole region of human chr. 6 of NA18570 (2.2x), NA18576 (1.8x), NA18592 (2.3x), and NA18944 (2.3x) were used for additional experiments for low-coverage data. Table 3 describes the comparative performance results of the four methods. The overall FNR for CNV_SS is between 17.31% and 41.08%, the FPR between 2.12% and 3.31% with relatively low coverage data. It can be seen that FNR and FPR may decrease as the level of read coverage increases. However, the performance of the proposed method has low dependency on the level of the read coverage. The results show that our method has fairly good FNR and FPR even at a very low level of read coverage (1.8-2.3x), and the proposed method outperforms the other methods by as much as 70.98% to 85.80%.

**Table 3:** Performance comparison of four methods on chromosome 6 sequence data of six individuals at relatively low coverage

**Method**	**NA18576 (1.8x)**		**NA18511 (1.8x)**		**NA18570 (2.2x)**		**NA18592 (2.3x)**		**NA18944 (2.3x)**		**NA10851 (5.6x)**	
	**FPR(%)**	**FNR(%)**	**FPR(%)**	**FNR(%)**	**FPR(%)**	**FNR(%)**	**FPR(%)**	**FNR(%)**	**FPR(%)**	**FNR(%)**	**FPR(%)**	**FNR(%)**
CNV_SS	2.37	39.89	2.12	41.08	2.36	23.79	3.31	36.77	2.48	37.24	2.77	17.31
CNV_shape	9.25	46.51	9.39	45.32	12.96	44.28	9.38	57.28	17.47	38.72	0.92	24.94
CNV-seq	0.12	68.84	0.13	86.29	0.11	81.99	0.09	80.86	0.10	82.78	0.07	75.17
CNV-seq	0.77	46.36	0.56	57.68	0.52	54.53	0.49	61.03	0.78	46.32	0.54	40.00
(modified)												

## Conclusion

A new method to detect CNVs based on scale-space filtering, called CNV_SS, is proposed. CNV_SS proceeds in two stages: up and down stages. In the up stage, read coverage data are transformed into a scale-space image with several layers by Gaussian convolution, and then the finger print map is obtained from the zero-crossing points of the second-order derivatives of the scale-space image per layer with increasing *σ*. In the down stage, baselines and intervals of each layer are calculated using the mean and the standard deviation of the read coverage data for each layer with decreasing *σ*. The intervals are the regions of the input sequence where CNV gains or losses are detected. The exact positions and the types of the CNV gains or losses are decided by the intervals and the baselines of each of the layers.

To verify the performance of this method, experiments using simulated data and real human data were undertaken. The simulated data with average coverage C (0.1x ≤C ≤10x) and error rate E (≤10%) were produced using contig NT_077531.3 of chr. 8 of NCBI Build 36.3 by inputting structural variations reported to the DGV, such as SNPs, indels, and CNVs, into its random positions. When the E error rate was fixed at 2% in the experiments using simulated data, the results showed that FNR and FPR decrease as the read coverage level increases, and stay in the range of 1.27% to 2.43% and 1.14% to 2.44%, respectively, even at a relatively low coverage (0.5x ≤C ≤2x). Moreover, in the experiments using simulated data with average coverage C (=1x, 3x) and the E error rate increasing by 1% within the range of 1% to 10%, the results yielded FPRs of 0.98% to 3.70% and FNRs of 1.25% to 4.25% at C=1x, and FPRs of 0.63% to 1.78% and FNRs of 0.92% to 2.56% at C=3x. The result suggests that CNV_SS can be very robust in error-prone environments, and the effect of errors can be reduced as the read coverage level increases. The experiments using simulated data discovered the relation between the scope of standard deviation and accuracy (FPR, FNR), which should be considered in scale-space filtering, according to the change of value in the C coverage rate and the E error rate.

In the experiments with real human data, pair-end reads of six hapmap samples, namely, NA10851 (5.6x), NA18511 (1.8x), NA18570 (2.2x), NA18576 (1.8x), NA18592 (2.3x), and NA18944 (2.3x), downloaded from the 1000 Genome Project website, were used. The FPRs and FNRs by the proposed method CNV_SS were evaluated on the basis of the CNV database of the DGV and then compared with conventional methods such as CNV_shape, CNV-seq, and modified CNV-seq. The proposed method gave a relatively similar output of FPR (≤3.31) with the conventional methods, whereas in FNR, the proposed method was found to be much more effective than the conventional methods, by 3.82% at the least and 76.97% at the most. These results show that CNV_SS can find CNVs effectively, using relatively low-coverage data, and also can find various CNVs regardless of their size.

## Competing interests

The authors declare that they have no competing interests.

## Authors’ contributions

JL designed algorithms and performed experiments and analysis. BK, EL and JY designed algorithms, performed analysis, and drafted the manuscript. All authors read and approved the final manuscript.
